# Prognostic value of the pan-immune-inflammation value for mortality in sepsis-induced coagulopathy: a Medical Information Mart for Intensive Care study

**DOI:** 10.1016/j.rpth.2025.103257

**Published:** 2025-11-17

**Authors:** Jian-Yue Yang, Li-Li Li, Su-Zhen Fu

**Affiliations:** Department of Critical Care Medicine, Xingtai People’s Hospital, Hebei, China

**Keywords:** disseminated intravascular coagulation, external validation, machine learning, pan-immune-inflammation value, prediction, sepsis

## Abstract

**Background:**

Sepsis is a life-threatening condition characterized by organ dysfunction caused by a dysregulated host response to infection. Its associated coagulopathy, known as sepsis-induced coagulopathy (SIC), significantly increases mortality risk. The pan-immune-inflammation value (PIV), a composite biomarker reflecting systemic immune and inflammatory status, has been linked to prognosis in various diseases.

**Objectives:**

This study aimed to evaluate the prognostic significance of PIV in patients with SIC and to develop predictive models accordingly.

**Methods:**

This retrospective study utilized data from the Medical Information Mart for Intensive Care IV database and included 4554 patients diagnosed with sepsis. Patients were stratified into high- and low-PIV groups based on the median PIV, and clinical characteristics were compared between groups. Kaplan–Meier survival analysis and Cox regression were employed to assess the association between PIV and patient outcomes. Least absolute shrinkage and selection operator regression was used to identify key variables for constructing a nomogram model. Additionally, machine learning algorithms, including random forest, were applied to build and validate predictive models.

**Results:**

Patients in the high-PIV group had significantly higher 30-day and 90-day mortality rates. Kaplan–Meier analysis showed that patients with lower PIVs had markedly better survival, and a nonlinear positive correlation was observed between PIV and mortality risk. Least absolute shrinkage and selection operator regression identified 8 key variables, including Acute Physiology Score III, lactate, red cell distribution width, mean corpuscular volume, acute kidney injury, and continuous renal replacement therapy. The nomogram based on these variables achieved areas under the receiver operating characteristic curve of 0.84 and 0.87 in the training and validation cohorts, respectively. Among machine learning models, the random forest algorithm exhibited the best predictive performance, with areas under the curve of 0.837 and 0.947 in the training and validation sets, respectively. External validation using a real-world cohort from Xingtai People’s Hospital further confirmed the association between elevated PIV and increased mortality and SIC, with consistent survival trends and nonlinear patterns observed in both Kaplan–Meier and restricted cubic spline analyses.

**Conclusion:**

To our knowledge, this study is the first to incorporate PIV into the prognostic assessment of patients with SIC. The development of a visual nomogram and machine learning-based models provides clinicians with practical tools for early identification of patients at high risk for SIC, potentially aiding in the optimization of treatment strategies.

## Essentials

• Sepsis-induced coagulopathy significantly increases the risk of mortality.
• Domestic and international databases were combined to assess pan-immune-inflammation value (PIV).
• Patients with high PIV had higher 30-day and 90-day mortality.
• PIV enables bedside early screening for high risk of sepsis-induced coagulopathy.

## 1 Introduction

According to the Third International Consensus Definitions for Sepsis and Septic Shock (Sepsis-3) [1], sepsis is defined as life-threatening organ dysfunction caused by a dysregulated host response to infection. Infection, particularly when it progresses to sepsis, induces widespread disturbances across multiple organ systems, including activation of the coagulation cascade. Coagulopathy is frequently observed in patients with sepsis, ranging from subclinical abnormalities to overt disseminated intravascular coagulation (DIC). In 2017, the Scientific and Standardization Committee of the International Society on Thrombosis and Haemostasis (ISTH) proposed the diagnostic criteria for sepsis-induced coagulopathy (SIC), aiming to facilitate early identification of DIC [2]. It has been reported that coagulopathy significantly increases the risk of mortality among sepsis patients, and SIC is associated with prolonged hospital and intensive care unit (ICU) stays, as well as higher mortality rates [3].

Systemic inflammation and immune dysregulation are considered key mechanisms in the pathophysiology of sepsis. Biomarkers reflecting immune and inflammatory status play a critical role in stratifying patient prognosis [4,5]. The pan-immune-inflammation value (PIV), which integrates several commonly used indicators of systemic inflammation, has emerged as a comprehensive index to assess both immune and inflammatory conditions [6]. Recent studies have demonstrated that elevated PIV is closely associated with adverse outcomes in hypertension [7], malignancies [8,9], cardiovascular diseases [10,11], and renal disorders [12]. Furthermore, PIV has shown predictive value for prognosis in patients with sepsis [6] and septic shock [13]. PIV incorporates peripheral neutrophil, platelet, monocyte, and lymphocyte counts, all of which have been implicated as key mediators in the pathophysiological processes of sepsis and coagulation abnormalities.

Nomograms, widely recognized as effective graphical tools for individualized risk estimation, have been increasingly applied in clinical prognostic research. Building on this framework, we hypothesized that elevated PIV would be independently associated with higher mortality among patients with SIC. To test this hypothesis, we leveraged a large-scale clinical database and contemporary statistical methodologies to investigate the prognostic relevance of PIV in SIC, identify key risk factors, and construct a novel nomogram model [14,15]. Furthermore, advances in machine learning (ML)—a pivotal subfield of artificial intelligence—offer powerful means of uncovering hidden patterns and modeling complex relationships within large datasets [16]. ML techniques have been shown to improve the early detection and diagnostic accuracy of SIC, support the development of personalized therapeutic strategies, and ultimately enhance clinical outcomes [17]. To further increase interpretability, the SHapley Additive exPlanations (SHAP) algorithm was employed, as it quantifies the contribution of each predictor to model outputs, thereby clarifying both the positive and negative effects of individual features on patient prognosis [18].

## 2 Methods

### 2.1 Data source

All data used in this study were extracted from the Medical Information Mart for Intensive Care IV (MIMIC-IV, v2.2) database, an openly accessible resource developed by the Laboratory for Computational Physiology at the Massachusetts Institute of Technology [19]. This database contains comprehensive demographic, clinical, laboratory, and outcome data for over 70,000 patients admitted to the Beth Israel Deaconess Medical Center in Boston, Massachusetts between 2008 and 2019. The richness and granularity of the dataset make it a valuable resource for clinical and epidemiological research.

In addition to the primary dataset from the MIMIC-IV database, we conducted external validation using an independent cohort from the ICU of Xingtai People’s Hospital, a tertiary teaching hospital in China. This cohort included patients with SIC diagnosed using the same Sepsis-3 and SIC criteria, and relevant clinical, laboratory, and outcome data were extracted using consistent definitions and preprocessing pipelines. The same PIV stratification method and survival analyses were applied to ensure comparability.

**Figure 1:**
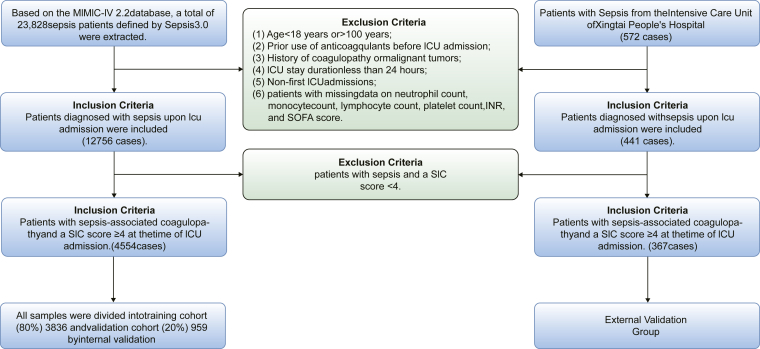
Data retrieval and model development. ICU, intensive care unit; INR, international normalized ratio; MIMIC-IV, Medical Information Mart for Intensive Care IV; SIC, sepsis-induced coagulopathy.

Patients were included in the study based on the following criteria: (1) age ≥18 years; (2) ICU stay ≥24 hours; and (3) diagnosis of sepsis upon ICU admission, according to the Sepsis-3 definition. Exclusion criteria were as follows: (1) patients aged <18 or >100 years; (2) history of anticoagulant therapy prior to ICU admission; (3) known coagulopathy or malignancy; (4) ICU length of stay <24 hours; (5) repeat ICU admissions; (6) missing values for key variables including neutrophil count, monocyte count, lymphocyte count, platelet count, international normalized ratio (INR), or Sequential Organ Failure Assessment (SOFA) score; and (7) patients with an SIC score <4 (Figure 1).

The first author, J.-Y.Y. (certification no. 62316152), was granted access to the MIMIC-IV database and conducted this research in accordance with the data use agreement. The external validation dataset was obtained from the ICU records of Xingtai People’s Hospital in Hebei, China, ensuring consistency in patient population and data structure for cross-cohort comparison.

### 2.2 Data extraction

Clinical data were extracted from the MIMIC-IV (v2.2) database using PostgreSQL and Navicat 16.1.12 database management systems. The dataset was randomly divided into a training set (80%) and a validation set (20%) via cross-validation. The extracted variables included: (1) demographic information; (2) clinical outcomes assessed at 30 and 90 days post-ICU admission; (3) severity scores, including the Acute Physiology Score III (APSIII) and the SOFA score; (4) vital signs and laboratory indicators recorded on the first day of ICU admission, including the lowest values of systolic blood pressure, diastolic blood pressure, heart rate, oxygen saturation, and body temperature, and laboratory parameters, including red blood cell count, red cell distribution width (RDW), mean corpuscular volume (MCV), white blood cell count, platelet count, INR, prothrombin time, activated partial thromboplastin time, lactate, chloride, anion gap, calcium, blood urea nitrogen, sodium, glucose, and creatinine; (5) comorbidities, including myocardial infarction, congestive heart failure, peripheral vascular disease, cerebrovascular disease, chronic obstructive pulmonary disease, diabetes, hypertension, and acute kidney injury (AKI); and (6) therapeutic interventions, including the administration of vasopressors, continuous renal replacement therapy (CRRT), and mechanical ventilation.

### 2.3 Definitions

In this study, SIC was defined according to the diagnostic criteria proposed in 2017 by the Scientific and Standardization Committee on DIC of the ISTH. The SIC scoring system comprises the following components: platelet count (100-150 × 10^9^/L = 1 point; <100 × 10^9^/L = 2 points), INR (1.2-1.4 = 1 point; >1.4 = 2 points), and SOFA score, representing organ dysfunction, was incorporated into the SIC scoring system and calculated based on the sum of 4 components: respiratory, hepatic, cardiovascular, and renal subscores (score of 1 = 1 point; score ≥2 = 2 points). A total SIC score ≥4, with the platelet count and INR components contributing at least 2 points, was required for a diagnosis of SIC.

PIV was calculated using the following formula:

$$\text{PIV}=\frac{\text{Neutrophils}\times \text{Platelets}\times \text{Monocytes}}{\text{Lymphocytes}}$$

All components were measured in units of 10^3^ cells/mm^3^.

### 2.4 Statistical analysis

All statistical analyses were performed using IBM SPSS Statistics Version 29.0 and R Version 4.4.2. Given the skewed distribution of PIV, a log_2_ transformation was applied prior to analysis. The median PIV was used as the cutoff to stratify patients into low- and high-PIV groups. The Kolmogorov–Smirnov test was used to assess the normality of variable distributions. For normally distributed continuous variables, comparisons between groups were performed using unpaired Student’s *t*-tests, and results are reported as mean ± SD. For nonnormally distributed variables, the Kruskal–Wallis test was used, with results expressed as median and IQR. Categorical variables are expressed as percentages and were compared using the chi-squared test or Fisher’s exact test, as appropriate. During the data extraction phase, 73 routinely used and easily obtainable clinical laboratory variables were collected. Multiple imputation was employed to handle missing data, excluding variables with ≥30% missing values. A total of 52 variables were retained and imputed, resulting in 5 imputed datasets. The dataset was then randomly divided, with 80% assigned to the training set and the remaining 20% to the validation set. The same procedure was applied to the external validation dataset.

Kaplan–Meier survival analysis was employed to assess 30-day and 90-day survival in patients with SIC with different PIVs. Survival differences were visualized, and hazard ratios (HRs) with corresponding 95% CIs were calculated. Comparisons between survival curves were conducted using the log-rank test.

To examine the impact of covariates on 30-day survival, Cox proportional hazards regression models were constructed. Model 1 was unadjusted (crude). Model 2 adjusted for sex and age. Model 3 further adjusted for SOFA and APSIII scores. Model 4 included additional adjustments for comorbidities and interventions, including myocardial infarction, congestive heart failure, peripheral vascular disease, cerebrovascular disease, chronic pulmonary disease, diabetes, hypertension, AKI, use of vasoactive drugs, mechanical ventilation, and CRRT.

Variable selection and regularization were performed using least absolute shrinkage and selection operator (LASSO) regression. Based on the training cohort, we initially included all 57 variables and applied 10-fold cross-validation to optimize the penalty parameter (λ). The optimal λ value of 0.00197 was selected by minimizing the binomial deviance. The selected variables were then used to construct a prognostic nomogram for patients with SIC. Model performance was evaluated using receiver operating characteristic (ROC) curves and the area under the curve (AUC). Clinical utility and net benefit of the nomogram were assessed via decision curve analysis in both the training and validation cohorts.

Based on the selected variables, 6 ML algorithms were developed to predict patient prognosis: decision tree, random forest, naïve Bayes, support vector machine, logistic regression, and gradient boosting machine. Predictive performance was evaluated by plotting ROC curves and calculating AUC, sensitivity, specificity, accuracy, F1 score, positive predictive value, and negative predictive value. To provide model interpretability, SHAP was used to generate summary and feature importance plots. *P* < .005 was considered statistically significant.

## 3 Results

### 3.1 Comparison of baseline clinical characteristics

A total of 4554 patients were included in the analysis and stratified into 2 groups based on the median PIV of 12: a low-PIV group (PIV < 12, *n* = 2261) and a high-PIV group (PIV ≥ 12, *n* = 2293). Baseline clinical characteristics were compared between the 2 groups (Table 1). No significant difference was observed in sex distribution. However, patients in the high-PIV group were older (71.05 vs 68.37 years, *P* < .001), had higher APSIII scores (53.00 vs 42.00, *P* < .001), and slightly lower SOFA scores (3.00 vs 4.00, *P* < .001).

**Table 1:** Comparison of baseline clinical characteristics.

Variables	Low PIV (*n* = 2261)	High PIV (*n* = 2293)	*P*
Demographics			
Sex			.26
Male	865 (38.26)	915 (39.90)	
Age, y	68.37 (58.77, 78.15)	71.05 (60.09, 81.23)	<.001
APSIII	42.00 (30.00, 61.00)	53.00 (41.00, 69.00)	<.001
SOFA score	4.00 (2.00, 5.00)	3.00 (2.00, 5.00)	<.001
ICU survival days	38.27 (6.35, 245.46)	29.62 (6.27, 194.26)	.24
Laboratory and physiological data			
Heart rate, bpm	82.00 (76.00, 97.00)	92.00 (79.00, 109.00)	<.001
Systolic blood pressure, mmHg	113.00 (101.00, 126.00)	116.00 (101.00, 132.00)	<.001
Diastolic blood pressure, mmHg	61.00 (53.00, 70.00)	64.00 (54.00, 76.00)	<.001
Respiratory rate, breaths/min	16.00 (14.00, 21.00)	20.00 (16.00, 24.00)	<.001
Temperature, °C	36.56 (35.90, 37.00)	36.78 (36.39, 37.17)	<.001
Blood oxygen saturation, %	100.00 (97.00, 100.00)	97.00 (94.00, 100.00)	<.001
International normalized ratio	1.50 (1.30, 1.70)	1.60 (1.50, 2.20)	<.001
prothrombin time, s	16.30 (14.60, 18.30)	18.00 (15.90, 23.40)	<.001
Partial thromboplastin time, s	32.30 (28.20, 38.70)	34.30 (29.60, 42.40)	<.001
Chloride, mmol/L	108.00 (104.00, 111.00)	104.00 (99.00, 108.00)	<.001
Anion gap, mmol/L	13.00 (11.00, 16.00)	15.00 (13.00, 18.00)	<.001
Calcium, mg/dL	8.00 (7.60, 8.50)	8.10 (7.50, 8.60)	.08
Blood urea nitrogen, mg/dL	18.00 (13.00, 28.00)	26.00 (17.00, 46.00)	<.001
Glucose, mg/dL	121.00 (104.00, 147.00)	129.00 (103.00, 170.00)	<.001
Creatinine, mg/dL	0.90 (0.70, 1.30)	1.30 (0.90, 2.10)	<.001
Monocytes, %	3.20 (2.00, 4.90)	5.00 (3.30, 7.30)	<.001
Neutrophils, %	75.80 (66.40, 82.00)	84.90 (79.00, 89.60)	<.001
Lymphocytes, %	16.60 (11.50, 23.10)	6.20 (3.80, 9.90)	<.001
Neutrophils, absolute, ×10^9^/L	6.98 (4.07, 10.22)	11.60 (7.70, 16.62)	<.001
Monocytes, absolute, ×10^9^/L	0.28 (0.16, 0.46)	0.66 (0.42, 1.02)	<.001
Lymphocytes, absolute, ×10^9^/L	1.46 (0.84, 2.19)	0.83 (0.50, 1.30)	<.001
Eosinophils, absolute, ×10^9^/L	0.07 (0.01, 0.14)	0.02 (0.00, 0.09)	<.001
Basophils, absolute, ×10^9^/L	0.02 (0.00, 0.03)	0.02 (0.00, 0.04)	.35
White blood cells, ×10^9^/L	9.50 (5.90, 13.30)	13.60 (9.50, 19.00)	<.001
Eosinophils, %	0.80 (0.10, 1.50)	0.10 (0.00, 0.70)	<.001
Red cell distribution width, %	14.20 (13.20, 15.70)	15.20 (14.00, 17.00)	<.001
Red blood cells, ×10^12^/L	3.06 (2.65, 3.56)	3.44 (2.93, 4.00)	<.001
Platelets, ×10^9^/L	110.00 (78.00, 136.00)	166.00 (123.00, 254.00)	<.001
Mean corpuscular volume, fL	92.00 (88.00, 96.00)	92.00 (87.00, 96.00)	.29
Hemoglobin, g/dL	9.40 (8.10, 10.80)	10.20 (8.70, 11.90)	<.001
Hematocrit, %	28.00 (24.40, 32.30)	31.60 (27.00, 36.20)	<.001
Lactate, mmol/L	1.50 (1.10, 2.20)	1.80 (1.20, 2.90)	<.001
log_2_(PIV)	10.60 (9.80, 11.30)	13.40 (12.60, 14.30)	<.001
Outcome			
In-hospital mortality			<.001
Death	1793 (78.19)	1967 (87.00)	
ICU expire flag			<.001
Death	1908 (83.21)	2048 (90.58)	
ICU outcome at 30 d			<.001
Death	1670 (72.83)	1929 (85.32)	
Hospital outcome at 90 d			<.001
Death	1478 (64.46)	1836 (81.20)	
Comorbidity			
Myocardial infarction	381 (16.85)	492 (21.46)	<.001
Congestive heart failure	627 (27.73)	996 (43.44)	<.001
Peripheral vascular disease	316 (13.98)	317 (13.82)	.88
Cerebrovascular disease	257 (11.37)	264 (11.51)	.88
Chronic pulmonary disease	513 (22.69)	654 (28.52)	<.001
Diabetes	619 (27.38)	775 (33.80)	<.001
Hypertension	670 (29.63)	551 (24.03)	<.001
Acute kidney injury	1747 (77.27)	1925 (83.95)	<.001
Treatment			
Used vasoactive drugs	1539 (68.07)	1390 (60.62)	<.001
Used ventilator	2073 (91.69)	2013 (87.79)	<.001
Used CRRT	142 (6.28)	216 (9.42)	<.001

Data are shown as *n* (%) or median (first quartile, second quartile).

APSIII, Acute Physiology Score III; CRRT, continuous renal replacement therapy; ICU, intensive care unit; PIV, pan-immune-inflammation value; SOFA, Sequential Organ Failure Assessment.

Physiological parameters including heart rate, respiratory rate, and body temperature were significantly elevated in the high-PIV group, whereas oxygen saturation was markedly lower (*P* < .001). Inflammation and coagulation markers also differed significantly; the high-PIV group exhibited elevated levels of INR, prothrombin time, and activated partial thromboplastin time, along with increased white blood cell and neutrophil counts, whereas both the percentage and absolute count of lymphocytes were significantly reduced (*P* < .001).

Regarding biochemical indicators, the high-PIV group had higher levels of blood urea nitrogen, creatinine, glucose, lactate, and anion gap, whereas chloride concentrations were lower (*P* < .001). Hematologic parameters also differed notably; patients in the high-PIV group had higher platelet counts, RDW, and hemoglobin concentrations (*P* < 0.001).

In terms of clinical outcomes, the high-PIV group exhibited significantly higher in-hospital mortality (87.00% vs 78.19%), ICU mortality, and 30-day and 90-day mortality rates (*P* < .001). Additionally, the prevalence of comorbidities such as myocardial infarction, congestive heart failure, chronic pulmonary disease, diabetes, and AKI was significantly higher in the high-PIV group (*P* < .001). Notably, patients receiving CRRT were more likely to have elevated PIV levels (*P* < .001).

In the external validation cohort from Xingtai People’s Hospital (Table 2), patients with PIV ≥ 12 (*n* = 172) presented a consistently heavier disease burden; they were older, carried significantly higher APS III scores, and exhibited an ICU mortality rate within 30 days of nearly 40%—substantially exceeding that of the low-PIV group. These findings mirror the pattern observed in the MIMIC-IV database, where elevated PIV was likewise associated with more frequent heart failure, AKI, and greater reliance on CRRT. Collectively, the parallel results from both cohorts underscore that a high PIV reliably signals more severe organ dysfunction and an markedly increased risk of early death among patients with SIC.

**Table 2:** Comparison of clinical baseline characteristics from external data

Variables	High PIV (*n* = 172)	Low PIV (*n* = 195)	*P*
Demographics			
Sex			.48
Male	105 (61.05)	126 (64.62)	
Age, y	73.32 (61.70, 80.64)	69.52 (58.80, 78.28)	.04
SOFA score	3.00 (2.00, 5.00)	3.00 (2.00, 5.00)	.47
APSIII	61.50 (49.00, 78.00)	46.00 (31.00, 61.50)	<.001
log_2_(PIV)	15.80 (15.60, 16.40)	11.80 (11.80, 11.90)	<.001
ICU outcome at 30 d			<.001
Death	68 (39.53)	31 (15.90)	
Comorbidity			
Heart failure	80 (46.51)	70 (35.90)	.04
Cerebrovascular disease	18 (10.47)	33 (16.92)	.07
Chronic pulmonary disease	50 (29.07)	48 (24.62)	.34
Diabetes	60 (34.88)	65 (33.33)	.75
Hypertensive	38 (22.09)	47 (24.10)	.65
Acute kidney injury	158 (91.86)	158 (81.03)	.003
Treatment			
Used vasoactive drugs	112 (65.12)	126 (64.62)	.92
Used ventilator	155 (90.12)	182 (93.33)	.26
Used CRRT	22 (12.79)	9 (4.62)	.005

Data are shown as *n* (%) or median (first quartile, second quartile).

APSIII, Acute Physiology Score III; CRRT, continuous renal replacement therapy; ICU, intensive care unit; PIV, pan-immune-inflammation value; SOFA, Sequential Organ Failure Assessment.

### 3.2 Kaplan–Meier survival analysis

**Figure 2:**
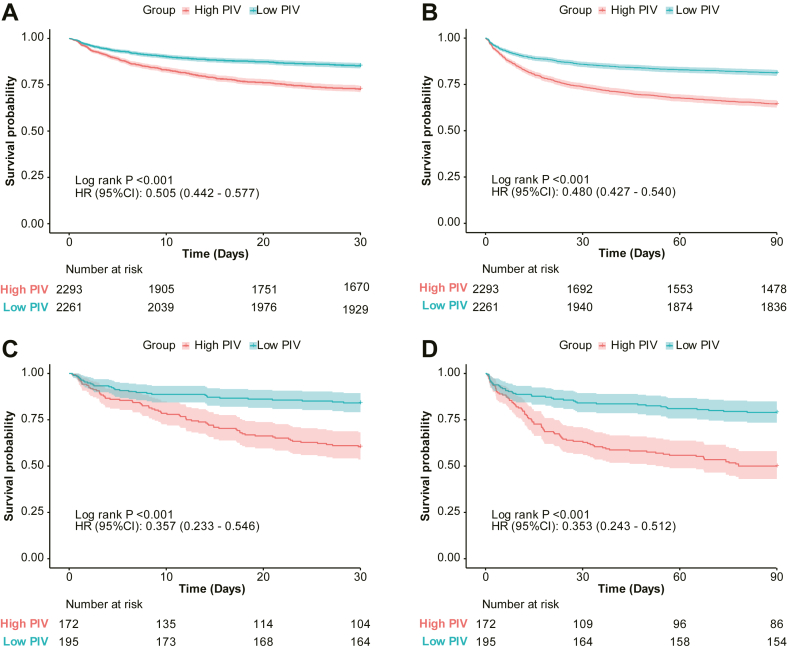
Kaplan–Meier survival curves for 30-day (A) and 90-day (B) mortality in high versus low-PIV groups from the MIMIC-IV cohort, and for 30-day (C) and 90-day (D) mortality from the external validation cohort. MIMIC-IV, Medical Information Mart for Intensive Care IV; PIV.

Kaplan–Meier survival analysis revealed that patients with lower PIV levels had significantly higher 30-day (Figure 2A) and 90-day (Figure 2B) survival rates compared with those with higher PIV levels, with differences reaching statistical significance (*P* < .001, log-rank test).

**Figure 3:**
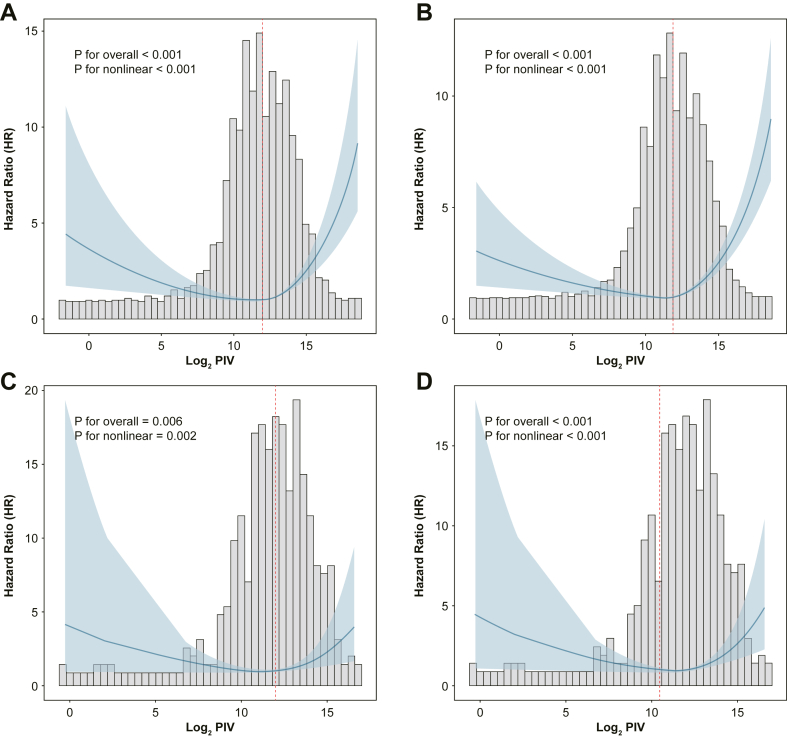
Restricted cubic spline models showing the nonlinear relationship between log_2_(PIV) and mortality in the MIMIC-IV cohort (A, B) and external validation cohort (C, D). MIMIC-IV, Medical Information Mart for Intensive Care IV; PIV, pan-immune-inflammation value.

Furthermore, restricted cubic spline (RCS) regression analysis (Figure 3) demonstrated that, in patients with SIC from both the MIMIC database and Xingtai People’s Hospital, log_2_(PIV) was significantly nonlinearly associated with adverse outcomes at 30 and 90 days. In the MIMIC cohort, both the 30-day and 90-day curves exhibited an initial increase followed by a plateau. Similarly, in the Xingtai cohort, the 30-day curve showed a comparable pattern, while the 90-day curve demonstrated a stronger association. Consistently across both centers, these results indicate a nonlinear positive correlation between elevated log_2_(PIV) and an increased short- and mid-term risk in patients with SIC.

### 3.3 Association between PIVs and short-term mortality

Table 3 illustrates the association between PIVs and survival risk across various adjustment models. The table reports HRs, 95% CIs, and *P* values, allowing for assessment of the prognostic significance of elevated PIVs and the corresponding statistical significance.

**Table 3:** Cox regression analysis for the relationship between PIV levels and clinical outcomes.

Variables	Model 1		Model 2		Model 3		Model 4	
	HR (95% CI)	*P*	HR (95% CI)	*P*	HR (95% CI)	*P*	HR (95% CI)	*P*
Group								
Low PIV	1.00 (Reference)		1.00 (Reference)		1.00 (Reference)		1.00 (Reference)	
High PIV	1.98 (1.73-2.26)	<.001	1.89 (1.66-2.17)	<.001	1.43 (1.25-1.64)	<.001	1.44 (1.25-1.66)	<.001

Model 1: Crude model. Model 2: Adjusted for sex and age. Model 3: Adjusted for sex, age, SOFA score, and APSIII. Model 4: Adjusted for sex, age, SOFA score, APSIII, myocardial infarction, congestive heart failure, peripheral vascular disease, cerebrovascular disease, chronic pulmonary disease, diabetes, hypertension, use of vasoactive drugs, ventilator use, CRRT, and AKI.

AKI, acute kidney injury; APSIII, Acute Physiology Score III; CRRT, continuous renal replacement therapy; HR, hazard ratio, PIV, pan-immune-inflammation value; SOFA, Sequential Organ Failure Assessment.

In the unadjusted model (model 1), patients in the high-PIV group had HR of 1.98. After adjustment for sex and age (model 2), the HR slightly decreased to 1.89. Upon further adjustment for SOFA and APSIII scores in model 3, the HR decreased to 1.43. In the fully adjusted model (model 4), which additionally accounted for comorbidities alongside sex, age, SOFA score, and APS III score, the HR remained at 1.44. All models yielded *P* values < .001, indicating that patients in the high-PIV group consistently exhibited significantly higher mortality risk compared to those in the low-PIV group.Although the HRs decreased progressively with adjustment for potential confounding factors, the elevated risk associated with high-PIV levels remained statistically significant across all models.

In the external validation cohort, patients with high-PIV levels also exhibited significantly higher 30-day and 90-day mortality, consistent with the findings from the MIMIC-IV dataset. Kaplan–Meier survival curves showed clear separation between high- and low-PIV groups (Figure 2C and D; log-rank *P* < .001). Additionally, RCS models demonstrated a similar nonlinear relationship between log_2_(PIV) and mortality risk (Figure 3C and D), reinforcing the threshold effect observed in the primary cohort.

### 3.4 LASSO regression analysis

A LASSO regression model was constructed based on the training cohort, initially incorporating 57 candidate variables. Given that certain variables, such as components of the SOFA score (vasopressor use, mechanical ventilation, creatinine, and bilirubin), INR, and platelet count, were used in the diagnosis of SIC or in the calculation of PIV (ie, neutrophils, lymphocytes, monocytes, and platelets), these variables were excluded to prevent potential multicollinearity and model interference.

The remaining 48 variables were subjected to 10-fold cross-validation, using binomial deviance as the evaluation metric. The optimal penalty parameter λ was determined to be 0.00197, which minimized model deviance.

**Figure 4:**
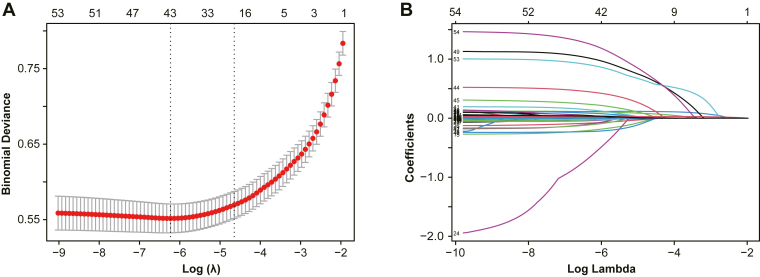
Least absolute shrinkage and selection operator (LASSO) coefficient path plot. (A) Cross-validation results of the LASSO logistic regression model. The x-axis represents the log(λ), and the y-axis indicates the mean cross-validation error. The solid line shows the trend of the mean error, and the dashed lines represent the upper and lower limits of the error. (B) Coefficient profiles of variables as a function of log(λ).

LASSO regression ultimately selected 8 key predictive variables spanning multiple clinical dimensions (Figure 4), including demographic factors (eg, age), comorbidities (eg, AKI), treatment interventions (eg, CRRT), severity scores (eg, APSIII), and laboratory indicators (eg, RDW, MCV, lactate, and log_2_-transformed PIV).

### 3.5 Nomogram development and internal validation

The variables selected by LASSO regression were incorporated into a multivariate logistic regression model to further assess their association with patient outcomes. As shown in Table 4, all included variables were significantly associated with adverse outcomes (*P* < .001 for all). The final model incorporated age, RDW, MCV, lactate, APSIII score, log_2_-transformed PIV, CRRT, and the presence of AKI.

**Table 4:** Multivariable logistic regression of nomogram-included predictors.

Variables	β	SE	Z	*P*	OR (95% CI)
Intercept	−10.94	0.88	−12.43	<.001	0.00 (0.00-0.00)
Age	0.01	0	3.72	<.001	1.01 (1.01-1.02)
RDW	0.1	0.02	5.14	<.001	1.11 (1.07-1.16)
MCV	0.02	0.01	3.21	<.001	1.02 (1.01-1.04)
Lactate	0.14	0.02	6.19	<.001	1.15 (1.10-1.20)
APSIII	0.03	0	13.13	<.001	1.03 (1.03-1.04)
log_2_(PIV)	0.11	0.02	4.72	<.001	1.12 (1.07-1.18)
CRRT	0.96	0.15	6.2	<.001	2.61 (1.93-3.53)
AKI	1.47	0.29	5.03	<.001	4.34 (2.45-7.69)

AKI, acute kidney injury; APSIII, Acute Physiology Score III; CRRT, continuous renal replacement therapy; MCV, mean corpuscular volume; OR, odds ratio; PIV, pan-immune-inflammation value; RDW, red cell distribution width.

The odds ratios (ORs) and 95% CIs for each variable were as follows: age (OR = 1.01, 95% CI: 1.01-1.02), RDW (OR = 1.11, 95% CI: 1.07-1.16), MCV (OR = 1.02, 95% CI: 1.01-1.04), lactate (OR = 1.15, 95% CI: 1.10-1.20), APSIII score (OR = 1.03, 95% CI: 1.03-1.04), log_2_-transformed PIV (OR = 1.12, 95% CI: 1.07-1.18), CRRT (OR = 2.61, 95% CI: 1.93-3.53), and presence of AKI (OR = 4.34, 95% CI: 2.45-7.69). Each of these variables was identified as an independent risk factor for adverse prognosis, with risk increasing incrementally per unit increase in the predictor.

**Figure 5:**
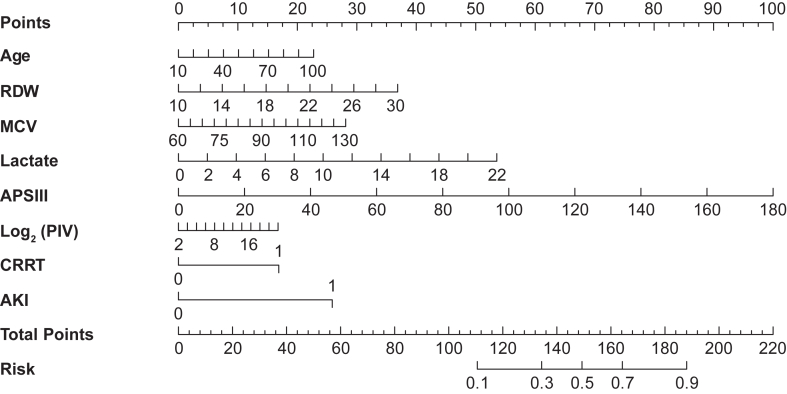
Prognostic nomogram for sepsis-induced coagulopathy outcomes. AKI, acute kidney injury; APSIII, Acute Physiology Score III; CRRT, continuous renal replacement therapy; MCV, mean corpuscular volume; PIV, pan-immune-inflammation value; RDW, red cell distribution width.

Based on these variables, a prognostic nomogram was constructed to predict patient outcomes (Figure 5). This model quantitatively integrated the contribution of each factor, enabling individualized risk prediction and offering a practical tool to support clinical decision making. The predictive model developed in this study demonstrated strong discrimination, good calibration, and high clinical applicability in both the training and validation cohorts.

**Figure 6:**
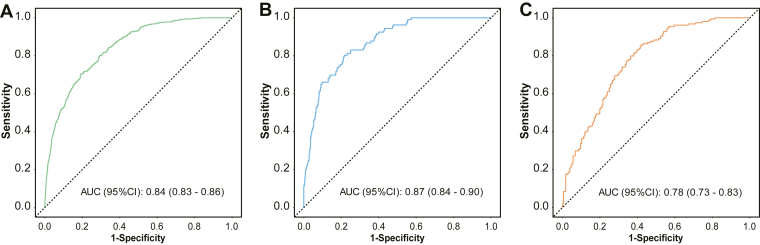
Receiver operating characteristic curves for the nomogram model in the (A) training set, (B) validation set, and (C) external validation cohort. AUC, area under the curve.

In terms of discrimination, ROC curve analysis showed an AUC of 0.84 (95% CI: 0.83-0.86) in the training cohort and 0.87 (95% CI: 0.84-0.90) in the validation cohort (Figure 6), indicating strong predictive performance of the nomogram.

**Figure 7:**
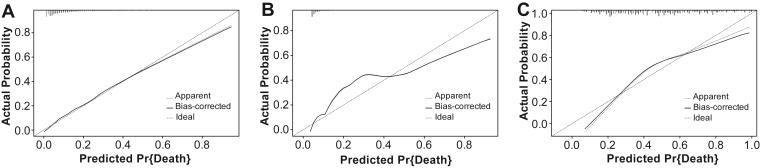
Calibration plots of the nomogram in the (A) training set, (B) validation set, and (C) external validation cohort. Pr, probability.

Calibration analysis further supported the model’s reliability. In the training cohort, the bias-corrected calibration curve closely aligned with the ideal reference line (Figure 7), reflecting good model fit. Similar consistency between predicted probabilities and actual outcomes was observed in the validation cohort, further confirming the model’s robustness.

**Figure 8:**
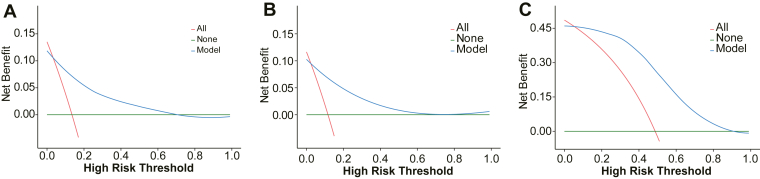
Decision curve analysis for the nomogram in the (A) training set, (B) validation set, and (C) external validation cohort.

Decision curve analysis was performed to assess the clinical utility of the model across a range of threshold probabilities. As illustrated in Figure 8, the nomogram yielded high net benefit across commonly used decision thresholds in both the training and validation cohorts, indicating strong potential for clinical application.

### 3.6 Comparison of ML model performance

**Figure 9:**
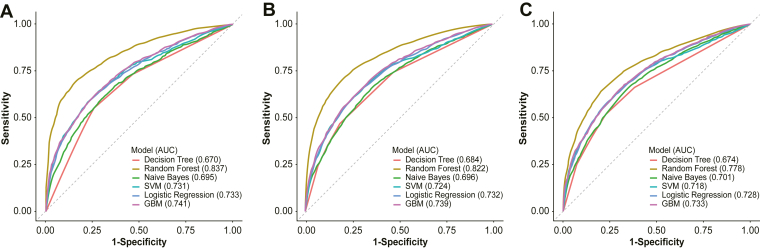
Receiver operating characteristic curves of the machine learning model in the (A) training set, (B) validation set, and (C) external validation set. AUC, GBM, gradient boosting machine; SVM, support vector machine.

Six ML models were developed using the training cohort, and their detailed performance metrics in evaluating the predictive value of PIV and other features for outcomes in patients with SIC are summarized in Table 5. The discriminative performance of each model, as assessed by ROC curve analysis, is shown in Figure 9A.

**Table 5:** Performance metrics of the ROC curves for machine learning models in the training set.

Models	Accuracy	Sensitivity	Specificity	PPV	NPV	Recall	F1 score
Random forest	0.83	0.85	0.82	0.84	0.83	0.85	0.845
GBM	0.74	0.76	0.73	0.75	0.74	0.76	0.755
SVM	0.73	0.75	0.72	0.74	0.73	0.75	0.745
Logistic regression	0.73	0.74	0.73	0.73	0.73	0.74	0.735
Naïve Bayes	0.7	0.72	0.69	0.71	0.7	0.72	0.715
Decision tree	0.67	0.65	0.68	0.66	0.67	0.65	0.655

GBM, gradient boosting machine; NPV, negative predictive value; PPV, positive predictive value; ROC, receiver operating characteristic; SVM, support vector machine.

Among the 6 models, the random forest algorithm demonstrated the best predictive performance, achieving an AUC of 0.837, sensitivity of 0.85, recall of 0.85, and F1 score of 0.845. This was followed by the gradient boosting machine (AUC = 0.741), logistic regression (AUC = 0.733), support vector machine (AUC = 0.731), naïve Bayes (AUC = 0.695), and decision tree (AUC = 0.670). Moreover, results from the validation set (Figure 9B) indicated that the random forest model achieved good predictive performance, with an AUC of 0.822, while the externally validated random forest model demonstrated comparable performance with an AUC of 0.778.

**Figure 10:**
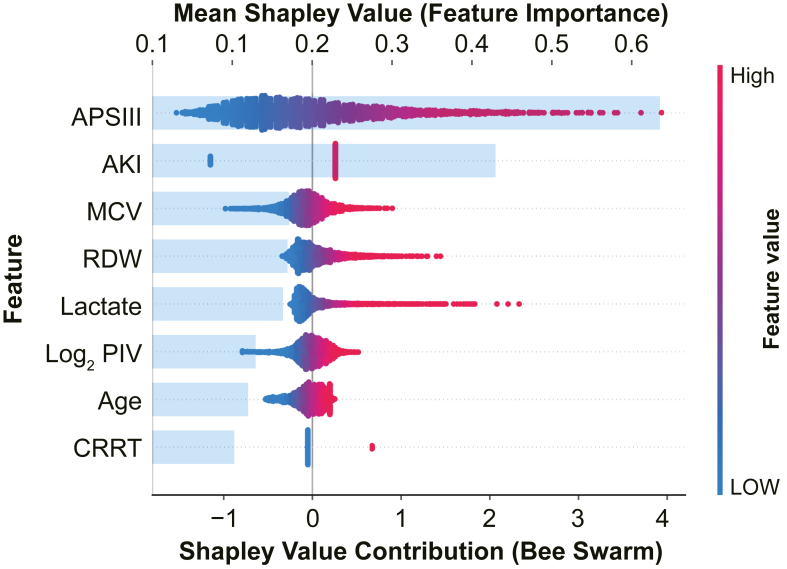
SHAP summary plot for the random forest model in the training set. AKI, acute kidney injury; APSIII, Acute Physiology Score III; CRRT, continuous renal replacement therapy; MCV, mean corpuscular volume; PIV, pan-immune-inflammation value; RDW, red cell distribution width; SHAP, SHapley Additive exPlanations.

Feature importance within the random forest model was evaluated using SHAP analysis. The SHAP summary plot (Figure 10) ranks features by their average importance (y-axis) and illustrates the relationship between individual feature values and SHAP values (x-axis), reflecting their contribution to the model’s predictions.For example, SHAP values for age were generally greater than zero, indicating that higher age was associated with an increased risk of mortality in patients with SIC.

## 4 Discussion

To our knowledge, this is the first study to integrate PIV into a prognostic model specifically tailored for patients with SIC, offering both clinical interpretability and ML-based prediction.

In this retrospective cohort study based on the large-scale MIMIC-IV database, we identified PIV as a novel immune-inflammatory marker that serves as a robust and independent predictor of adverse outcomes in patients with SIC. We developed and internally validated a prognostic model that integrates PIV with other clinical parameters. Our findings showed that elevated PIVs were significantly associated with increased 30-day and 90-day mortality, even after adjusting for multiple confounders. In addition, RCS analysis revealed a nonlinear positive relationship between log_2_(PIV) and mortality at both time points. Through LASSO regression, we identified 8 independent prognostic factors—log_2_(PIV), age, RDW, MCV, lactate, APSIII score, CRRT, and AKI—which were used to construct a nomogram for individualized risk estimation.

To better interpret the association between elevated PIV and adverse outcomes in SIC, it is important to consider the biological functions of its constituent cell types—neutrophils, lymphocytes, monocytes, and platelets—within the pathophysiological context of SIC. The PIV formula multiplies neutrophils, monocytes, and platelets—cells reflecting innate immune activation and procoagulatory activity—while dividing by lymphocytes to capture adaptive immunosuppression. This composite index integrates hyperinflammation, immune suppression, and coagulopathy, providing a more comprehensive reflection of SIC pathophysiology than simpler ratios such as neutrophil-to-lymphocyte ratio (NLR) or platelet-to-lymphocyte ratio (PLR). Compared with simpler ratios such as NLR or PLR, PIV offers several advantages. First, it integrates multiple immune and hematologic components—neutrophils, monocytes, and platelets—while accounting for lymphocyte-mediated adaptive immunosuppression, thereby simultaneously reflecting hyperinflammation, immune suppression, and coagulopathy. In contrast, NLR mainly captures the balance between innate and adaptive immunity [20], and PLR primarily reflects platelet-mediated inflammation and coagulation interactions [21,22]. Second, PIV provides a more comprehensive representation of SIC pathophysiology, which involves complex interactions among excessive innate immune activation, immunosuppression, endothelial injury, and microvascular thrombosis. Finally, PIV can be rapidly calculated at the bedside using routine complete blood counts without additional cost, facilitating early identification of high-risk patients and informing clinical decision making. Collectively, these advantages make PIV a superior biomarker for capturing the multifaceted immune-coagulatory derangements characteristic of SIC. Neutrophils and monocytes are integral components of the innate immune response and play key roles in amplifying inflammation and promoting thrombin generation, contributing to hallmark features of SIC such as endothelial injury and microvascular thrombosis [23,24].

In contrast, lymphopenia is a typical manifestation of sepsis-induced immunosuppression, resulting from apoptosis and impaired proliferation, and is strongly associated with poor prognosis [25]. Thrombocytopenia has also been linked to unfavorable outcomes in sepsis [26]. Platelets, in addition to their role in hemostasis, act as proinflammatory mediators and are crucial regulators of the immune response [27]. Activated platelets interact with neutrophils and monocytes, facilitate the formation of neutrophil extracellular traps, and promote the release of inflammatory cytokines [[28], [29], [30]]. Collectively, PIV integrates these cell types to reflect the overall immune-inflammatory state. An elevated PIV suggests immune dysregulation—characterized by excessive innate activation, adaptive immunosuppression, and coagulopathy—which aligns with the clinical trajectory of SIC from hyperinflammation to immune exhaustion and multiorgan dysfunction.

The interplay between inflammation, immune dysfunction, and coagulopathy has been well described in sepsis-related studies. Previous evidence has demonstrated that inflammatory ratios such as the NLR and PLR are associated with poor prognosis in sepsis [31]. First proposed by Fucà et al. in 2020 [32], PIV combines 4 critical immune cell counts and provides a more comprehensive perspective on systemic inflammation than indices such as the aggregate index of systemic inflammation. PIV has shown prognostic utility in various conditions, including malignancy, hypertension, and cardiovascular diseases [26]. Our study extends its application to the SIC population, filling a gap in the literature and supporting its potential as a surrogate marker for immune-coagulatory imbalance in this setting.

Unlike previous studies that primarily focused on the general sepsis population, our research targeted a specific subgroup of patients who met ISTH criteria for SIC. Even after adjusting for SOFA scores, APSIII scores, and multiple comorbidities, PIV remained an independent predictor of mortality (OR = 1.12, 95% CI: 1.07-1.18) [6]. Importantly, PIV can be rapidly calculated at bedside from routine complete blood counts without additional cost, making it feasible for early risk stratification in patients with SIC. Although our findings support its role as an early warning biomarker, we acknowledge that its prospective utility in guiding clinical decisions remains to be validated. Accordingly, we explicitly note that future multicenter prospective studies are warranted to assess whether serial PIV monitoring can enhance dynamic risk assessment and guide individualized interventions in SIC.

SHAP analysis further confirmed that PIV was among the most influential predictors in the ML model. Mechanistically, the proportional changes of the cellular components within PIV correspond to typical SIC pathophysiology—neutrophil-driven inflammation, lymphocyte depletion-induced immunosuppression, dysfunctional monocyte activity, and platelet activation-induced endothelial injury and microthrombi formation. Therefore, elevated PIV may reflect systemic immune-coagulatory derangement, and our findings support its potential use as an early warning biomarker in the clinical progression of SIC.

In addition to PIV, we identified APSIII score, lactate, RDW, MCV, AKI, and CRRT as independent predictors of mortality in SIC patients. APSIII and lactate are well-established markers of illness severity and impaired perfusion [13], while AKI and CRRT often indicate multiorgan failure and escalation of care, both associated with poor outcomes. RDW and MCV reflect red blood cell heterogeneity and oxygen metabolism dysfunction, suggesting possible metabolic disturbances during SIC progression. These findings highlight that accurate prognostication in SIC requires integrating inflammatory markers with indices of organ function, metabolic status, and comorbid conditions [33]. Furthermore, RCS modeling revealed a nonlinear association between PIV and mortality risk, suggesting that its predictive effect may become more pronounced above certain thresholds. Future research should explore the dynamic changes of PIV over time and evaluate its utility in guiding therapeutic strategies.

Moreover, potential population heterogeneity should be acknowledged. The primary cohort was derived from a US academic medical center, whereas the validation cohort was obtained from a Chinese tertiary hospital. Differences in healthcare systems, patient demographics, and clinical practice patterns may have influenced the observed outcomes. In addition, the prognostic model was constructed based only on baseline PIVs at ICU admission, and dynamic changes in PIVs over time were not assessed. Given that sepsis and SIC are highly dynamic processes, the absence of serial monitoring may limit the model’s ability to capture temporal variations in immune–inflammatory and coagulatory status. External validation in large, prospective, multicenter cohorts is also needed to improve the generalizability and clinical reliability of our model.

Several limitations of this study should be acknowledged. First, although we performed external validation using an independent cohort from Xingtai People’s Hospital, the primary analysis remains a single-center retrospective study based on the MIMIC-IV database, which may introduce selection and information biases. In addition, multiple imputation was conducted before train-test splitting, so imputation parameters were informed by the whole cohort and may have marginally biased the estimates, although we expect this impact to be small and nondirectional. Second, important biomarkers such as D-dimer and interleukin-6 were not included in the analysis, raising the possibility of residual confounding. Third, although variable selection and model interpretability were optimized using LASSO regression and SHAP algorithms, the model has yet to be validated in external populations. Finally, implementation of ML-based tools in real-world clinical settings remains challenging, requiring integration with electronic health record (EHR) systems and adequate training for healthcare providers.

Several limitations of this study should be acknowledged. First, this was a retrospective analysis based primarily on the MIMIC-IV database, with external validation performed in a single tertiary hospital in China. Although this design strengthens the robustness of our findings, the use of a single database and a single external cohort may still limit generalizability. Second, important biomarkers such as D-dimer and interleukin-6 were not available in either dataset, raising the possibility of residual confounding. Third, although variable selection and model interpretability were optimized using LASSO regression and SHAP algorithms, the model requires further validation in larger, prospective, multicenter cohorts. Moreover, potential population heterogeneity should be acknowledged: the primary cohort was derived from a US academic medical center, whereas the validation cohort was obtained from a Chinese tertiary hospital. Differences in healthcare systems, patient demographics, and clinical practice patterns may have influenced the observed outcomes. In addition, the prognostic model was constructed based only on baseline PIV values at ICU admission, and dynamic changes in PIV over time were not assessed. Given that sepsis and SIC are highly dynamic processes, the absence of serial monitoring may limit the ability of our model to capture temporal variations in immune–inflammatory and coagulatory status. Finally, implementation of machine learning-based tools in real-world clinical settings remains challenging, requiring integration with EHR systems and adequate training for healthcare providers. Future prospective multicenter studies incorporating longitudinal PIV monitoring are warranted to address these limitations.

To validate the robustness and generalizability of our findings, we conducted an external validation using an independent cohort from Xingtai People’s Hospital in China. Consistent with the results obtained from the MIMIC-IV database, patients with high PIV in the external cohort exhibited significantly higher 30-day and 90-day mortality, as illustrated by Kaplan–Meier survival curves. Additionally, RCS analysis demonstrated a similar nonlinear relationship between PIV and mortality in both cohorts. These findings confirm the predictive value of PIV across distinct populations and healthcare settings, supporting its potential applicability in diverse clinical environments. External validation not only enhances the credibility of our model but also paves the way for future multicenter studies and real-world implementation.

Despite these limitations, our study presents substantial clinical and methodological innovation. To our knowledge, this is the first study to incorporate PIV into an interpretable, visual predictive model specifically for SIC, combining traditional regression with ML and explainability techniques. The nomogram provides a simple and practical tool for bedside risk estimation, while SHAP plots assist in identifying individualized risk contributors. In practice, PIV could be used as an early screening marker upon ICU admission to support decisions regarding anticoagulation, immunomodulatory therapy, or escalation of care. The model also has the potential to be embedded into ICU EHR systems as a real-time decision support tool. Future prospective multicenter studies are needed to validate its broader applicability and to investigate how PIV dynamics correlate with treatment response.

## 5 Conclusion

This study demonstrated that elevated PIV is an independent predictor of both short-term and long-term adverse outcomes in patients with SIC. Based on this finding, we developed and internally validated a nomogram model for SIC risk prediction in critically ill patients. The model integrates several key clinical variables, including APSIII score, lactate, RDW, MCV, AKI, and CRRT, all of which were significantly associated with SIC occurrence and prognosis. By improving the accuracy of outcome prediction, this model may assist clinicians in the early identification of high-risk patients and the optimization of individualized treatment strategies, offering promising potential for clinical application. The consistent findings in an external real-world cohort further validate the applicability of PIV across diverse intensive care settings.
